# Egalitarian mixed-species bird groups enhance winter survival of subordinate group members but only in high-quality forests

**DOI:** 10.1038/s41598-020-60144-w

**Published:** 2020-03-04

**Authors:** Indrikis A. Krams, Severi Luoto, Tatjana Krama, Ronalds Krams, Kathryn Sieving, Giedrius Trakimas, Didzis Elferts, Markus J. Rantala, Eben Goodale

**Affiliations:** 1 Institute of Ecology and Earth Sciences, University of Tartu, Tartu, 51014 Estonia; 2 Latvian Biomedical Research and Study Centre, Rīga, 1067 Latvia; 3 Department of Zoology and Animal Ecology, Faculty of Biology, University of Latvia, Rīga, 1004 Latvia; 4 English, Drama and Writing Studies, University of Auckland, Auckland, 1010 New Zealand; 5 School of Psychology, University of Auckland, Auckland, 1010 New Zealand; 6 Department of Plant Health, Estonian University of Life Sciences, Tartu, 51014 Estonia; 7 Department of Wildlife Ecology, University of Florida, Gainesville, Florida 32611 United States; 8 Institute of Biosciences, Vilnius University, Vilnius, 10257 Lithuania; 9 Department of Botany and Ecology, Faculty of Biology, University of Latvia, Rīga, 1004 Latvia; 10 Department of Biology, University of Turku, Turku, 20014 Finland; 11 Turku Brain and Mind Centre, University of Turku, Turku, 20014 Finland; 12 Guangxi Key Laboratory of Forest Ecology and Conservation, College of Forestry, Guangxi University, Nanning, Guangxi 530004 China

**Keywords:** Behavioural ecology, Social evolution

## Abstract

Only dominant individuals have unrestricted access to contested resources in group-living animals. In birds, subordinates with restricted access to resources may respond to intragroup contests by acquiring extra body reserves to avoid periods of food shortage. In turn, higher body mass reduces agility and increases predation and mortality risk to subordinates. Birds often live in hierarchically organized mixed-species groups, in which heterospecific individuals are considered to substitute for conspecifics as protection against predators at a significantly reduced competition cost. Crested tits (*Lophophanes cristatus*) and willow tits (*Poecile montanus*) form mixed-species groups during the non-reproductive season that typically exhibit a nearly linear dominance hierarchy (‘despotic’ social structure) in which the highest ranking male willow tit is fourth in the overall hierarchy after the dominant male, female and subordinate juvenile crested tit, respectively. Much less frequently, ‘egalitarian’ dominance structures occur in which the adult willow tits rank second and the hierarchy is less steep, or linear. We present a rare long-term data set in which egalitarian flocks are common enough to assess the consequences of this simple change in hierarchy structure as well as a potential driver of the pattern. A comparison of individuals in the despotic mixed-species groups revealed a strong negative correlation between subcutaneous fat stores and dominance rank in the interspecific dominance hierarchy, whereas in egalitarian groups, subordinate willow tits had significantly lower fat reserves and they foraged in safer parts of the canopy than willow tits in despotic groups. Moreover, egalitarian groups exhibited markedly less within-group aggression, higher group cohesion and improved winter survival in both tit species. However, winter survival of birds in egalitarian groups was impaired relative to despotic groups in forests recently affected by industrial forestry. This suggests that the more egalitarian bird societies may best be adapted to less-disturbed environments.

## Introduction

### Non-breeding sociality in temperate passerines

The gregarious grouping of animals is a widely occurring biological phenomenon. Social individuals can assemble either in single-species or in mixed-species groups, with both types of groups being common in birds^1–3^. In some habitats, mixed-species bird groups dominate entire avifaunas^4,5^, although such groups are not often considered in the analysis of avian community structure^6^. Hierarchical social structure is a defining feature of animal groups (both mixed-species and single-species) where more dominant or aggressive individuals win contests over less aggressive members of a hierarchy. This can pose survival costs to subordinates^7^. At the same time, a major incentive for subordinates to join groups with hierarchical structure is gregariousness, which provides protection from predators. The “many eyes” hypothesis posits that the task of scanning the environment for predators is effectively shared by many individuals in larger groups^8–11^. Being a member of a larger group also dilutes the risk of attack to any individual^8,12–14^. The opportunity for both predation risk dilution and increased vigilance should encourage mixed-species group formation^6,8^, as should a reduction in competition for food, because interspecific competition for food is usually less severe than intraspecific competition^15–18^.

Birds in family Paridae (parids; tits, chickadees, and titmice) are well-studied passerines^19^ that habitually form mixed-species social groups during temperate zone winters^9^ (the non-breeding season). Field observations^20,21^ and experiments demonstrate that intragroup competition restricts access to food^22^ and to the safest and most preferred feeding sites in these groups^2,23,24^. Dominant individuals in these hierarchical groups have priority access to contested resources—a fact that often results in better winter survival of the dominant individuals^25–27^. In contrast to the survival advantages of dominants, subordinates usually pay for their membership in both single- and mixed-species groups by higher mortality rates^23,28–30^.

### Dominance, fat reserves, and survival in mixed-species groups

In mixed-species parid groups, heterospecifics of lower-ranking species are generally considered to substitute for conspecifics of a dominant species as protection against predators at a lower competition cost^8,31^. Reduced competition, however, does not make a subordinate individual’s food supply as predictable as a dominant’s and this, in turn, increases the starvation risk of subordinates. At high latitudes, subordinates respond to unpredictable nutritional conditions by acquiring and carrying extra body reserves (e.g., fat) as a buffer against periods of food shortage or high energy demands exacerbated by low ambient temperatures and/or pathogen-induced anorexia^32^. Indeed, dominants are most likely to take advantage of their higher rank in resource acquisition when temperatures drop^33,34^. Although more body reserves reduce the risk of starvation^33^, extra body mass also imposes a significant survival cost by increasing the risk of predation^35,36^.

Fattening strategies dependent on social status represent a group-specific cost to subordinates, including subordinate species in mixed-species groups. Krams^34^ documented significantly greater body reserves of heterospecific subordinates in mixed-species groups of dominant crested tit (*Lophophanes cristatus*) and subordinate willow tit (*Poecile montanus*) in winter. Body reserves gradually increased as the social rank decreased from adult crested tit males to the most subordinate willow tit juvenile females. In these mixed-species groups, social relationships were highly ‘despotic’ in that crested and willow tits formed nearly linear dominance hierarchies: crested tits were above willow tits during aggressive encounters over resources, the only exception being adult male willow tits that were above juvenile female crested tits^34^. Within each species, males dominate conspecific females and adults dominate juveniles^24,34^. At high ambient temperatures, these groups often split up temporarily into monospecific subgroups of an adult pair or juveniles^37^, suggesting that subordinates’ costs of socially imposed winter fattening may be significant in such despotic hierarchies. Warming-enhanced food availability (and reduced aggression from dominant crested tits) likely drives temporary mixed group dissolution, despite the costs of reduced collective vigilance for both subgroups.

### Can hierarchy structure influence subordinate survival?

Dominance hierarchies in general, and in parid mixed-species groups, can vary from very steep and linear (or ‘despotic’) where each individual dominates those below in every interaction, or more flat (‘egalitarian’) where individuals can experience reversals (wins or losses) in contests with dominants or subordinates^8^. In general, egalitarian groups may be larger than despotic ones with more diverse social connections that have been linked to long-term familiarity between individuals (within and between species)^38,39^. This suggests that competition in more egalitarian groups might be less severe due to more efficient communication among familiar individuals^15,40,41^. Moreover, the larger group size may improve winter survival in egalitarian groups through enhanced antipredator protection^15,40,42^.

Egalitarian crested and willow tit groups are unusual, comprising less than 20% of observed groups. Egalitarian crested and willow tit groups are distinctive in make-up, however. The adult willow tit males have a higher than usual rank (compared to despotic groups); they dominate both the juvenile crested tit (male and/or female) and the adult crested tit females (personal obs.). Despite the rarity of egalitarian groups, we present analyses of a long-term and detailed data set with sufficient sample size to address the consequences for egalitarian group members.

### Hypotheses, predictions and study design

**Figure 1:**
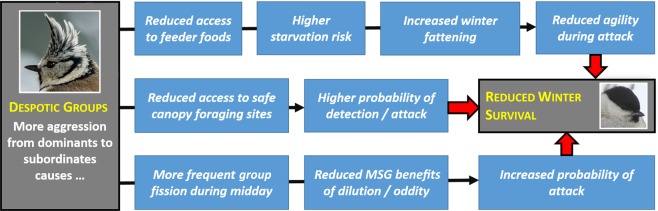
Hypothesized mechanisms underlying lower subordinate survival in despotic mixed-species groups (MSG) of crested and willow tits, relative to subordinate individuals in egalitarian mixed-species groups that experience reduced aggression from dominants. Specific predictions are that females and juveniles of both tit species (but especially willow tits) in despotic groups will experience lower survival via the combined effects of increased fattening, lower foraging heights and less time spent in safer mixed species groups—all resulting from higher aggression in despotic groups.

In order to test whether subordinates in egalitarian groups incur lower survival costs than their social equivalents in despotic groups, a known gradient of differential predation risk experienced by group participants is required. In our study system, predation risk varies with foraging location in single trees and is significantly higher for birds that forage lower in the canopy of mature trees^20,21,24,28^. If competition is lower in egalitarian groups, then we predict (1) that subordinate individuals should forage relatively higher in the canopy when they are members of egalitarian versus despotic groups. (2) Egalitarian groups should also exhibit greater coherence because subordinates need not escape their position in the system as much as they do in despotic groups. Further, (3) winter survival should be higher in egalitarian systems than in despotic ones (Fig. 1).

In this study, we compared despotic and egalitarian crested and willow tit groups. While observing despotic and egalitarian mixed-species groups, we measured the birds’ body mass index (BMI), the relative foraging height in the tree canopy, the number of aggressive encounters and group cohesion expressed as a proportion of time the two species forage together (indicating the intensity of competition). In addition to the predictions about higher foraging height, greater cohesion and greater survival in egalitarian groups discussed above, we also investigated the effect of land-use on this social system. It is known that in the forests where the adjacent land has been clear-cut, the birds are more exposed to predation^43,44^. Furthermore, because of higher levels of sunlight and direct exposure to wind, the parts of the forest close to the edge dry out, negatively affecting habitat quality^45^. Therefore, we tested whether the “edge effect” of clear-cut areas had any influence on winter survival of willow and crested tits in the two types of social groups.

## Results

### Dominance interactions

Group sizes were the same across all flocks and adult crested tit males dominated in both types of social units. The total number of aggressive interactions observed in egalitarian groups was significantly lower (7.38 ± 1.73 aggressive encounters hr^−1^) than that observed among members of despotic groups (12.71 ± 3.58 aggressive encounters hr^−1^; one way ANOVA, *F*_(1, 36)_ = 32.95, *P* < 0.0001, with group size constant across the groups, *n* = 60).

### Body reserves (BMI)

**Figure 2:**
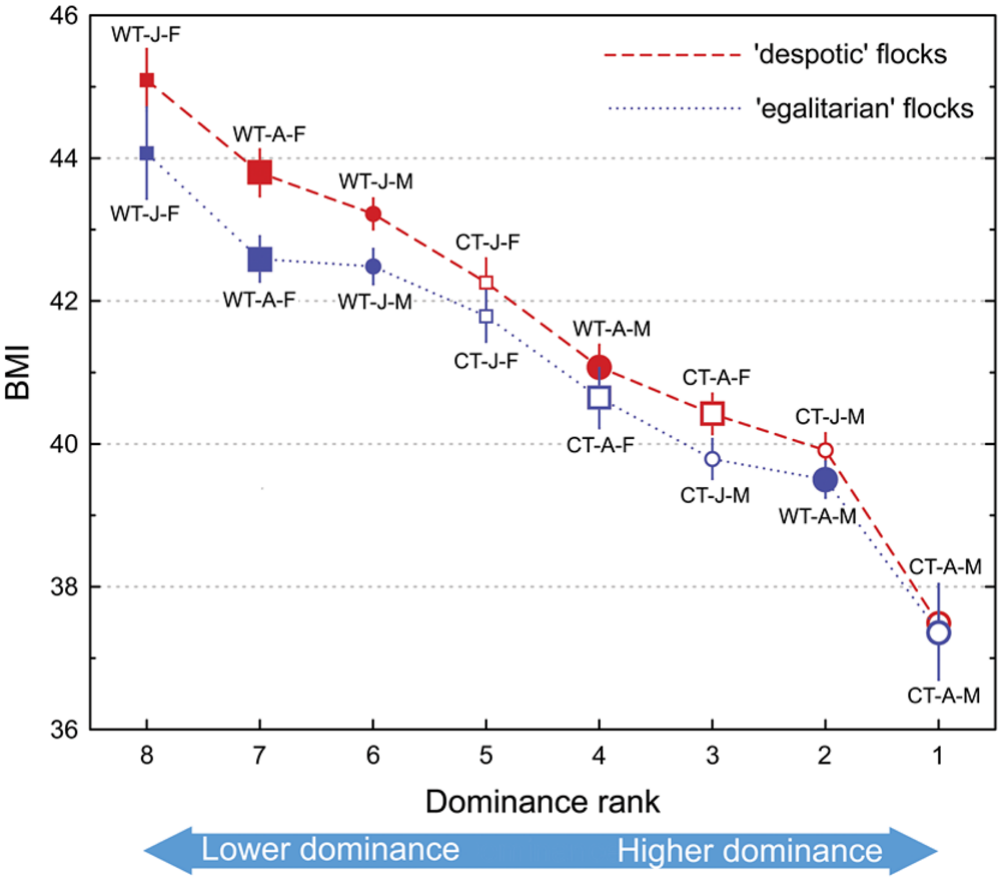
Average body mass index (BMI ± 95%CI) of crested tits (open symbols) and willow tits (closed symbols) plotted against their dominance rank (#1 as the highest rank) in the ‘despotic’ and ‘egalitarian’ mixed-species groups. Circles indicate males, squares: females. Large symbols show adults, small ones: juveniles. CT: crested tit; WT: willow tit; A: adult; J: juvenile; M: male; F: female.

While wing and tarsus length did not differ significantly between the two types of social groups (one-way ANOVA: all *P*s > 0.05), BMI varied as follows. We detected a strong negative correlation between the BMI of individuals of the two tit species and their dominance rank in the mixed-species groups (*r*_*s*_ = −0.93, *P* < 0.005; Fig. 2). The BMI of individuals in both species gradually decreased from juvenile females (highest BMI) to adult females, juvenile males and to adult males (lowest BMI). While the BMI of crested tits was similar between the two types of social groups (*t*-test: *t*(150) = 0.39, *P* = 0.7), willow tits exhibited significantly lower BMI in the egalitarian groups than in the despotic groups (*t*-test: *t*(150) = 4.04, *P* < 0.0001; Fig. 2). Specifically, the BMI of adult willow tit males was significantly lower in egalitarian groups (39.5 ± 0.52, mean ± *SD*) than in despotic groups (41.09 ± 0.68, mean ± *SD*: *t*(36) = 8.11, *P* < 0.0001, Fig. 2). The BMI of adult willow tit females was significantly lower in egalitarian groups (42.59 ± 0.64, mean ± *SD*) than in despotic groups (43.8 ± 0.7 mean ± *SD*; *t*(36) = 5.49, *P* < 0.001, Fig. 2). The BMI of willow tit juvenile males was also significantly lower in egalitarian groups (42.48 ± 0.52, mean ± *SD*) than in despotic groups (43.22 ± 0.47, mean ± *SD*), *t*(36) = 4.71, *P* < 0.001, Fig. 2). Finally, the BMI of juvenile willow tit females was also significantly lower in egalitarian groups (44.07 ± 1.29, mean ± *SD*) than in despotic groups (45.1 ± 0.93, mean ± *SD*; *t*(36) = 2.83, *P* = 0.008; Fig. 2).

### Group cohesion

**Figure 3:**
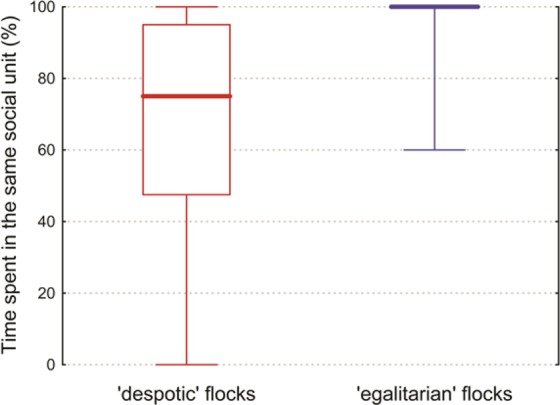
Proportion of time spent in the same social unit in the ‘despotic’ and ‘egalitarian’ mixed-species groups. The thick lines show the medians, the boxes: 25^th^–75^th^ percentiles, the whiskers: min–max values.

We found that all groups (both despotic and egalitarian) remained highly cohesive during the observations in the cold morning hours. However, time spent together in the same social unit at noon differed significantly between despotic and egalitarian groups (Mann-Whitney test, *U* = 64, *p* < 0.001). Most egalitarian groups did not split during the observation period at noon (median = 100%, *n* = 18). In contrast, in the despotic groups, willow tits spent less time in the same social unit with crested tits (median = 75%, *n* = 20; Fig. 3).

### Relative foraging height

**Figure 4:**
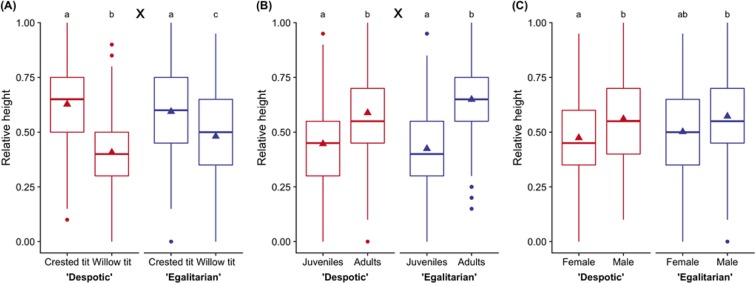
The relative foraging heights of willow and crested tits in the pine canopy as members of ‘despotic’ and ‘egalitarian’ mixed-species groups *vs* species (**A**), age **(B**), sex (**C**). Triangles indicate means, thick lines: medians, boxes: interquartile ranges (IQRs), whiskers: 1.5*IQRs, points: outliers. Letters indicate significant differences (*P* < 0.05); X indicates interactions between dominance systems, species and age groups.

Crested tits foraged significantly higher in the canopy both in despotic and egalitarian groups than willow tits (LMM, *F*_(1,1499)_ = 591.28, *P* < 0.001). However, there were significant interactions between species and social unit (LMM, *F*_(1,1499)_ = 61.66, *P* < 0.001; Fig. 4A). Most importantly, willow tits were observed foraging significantly higher in the canopy when in egalitarian groups than in despotic groups (Tukey contrasts, *P* < 0.02; Fig. 4A). Juvenile crested and willow tits foraged significantly lower than adult individuals (LMM, *F*_(1,1499)_ = 722.23, *P* < 0.001), although there were significant interactions between age and social unit (LMM, *F*_(1,1499)_ = 36.42, *P* < 0.001) (Fig. 4B), and between sex and age (LMM, *F*_(1,1499)_ = 10.83, *P* = 0.001). Females foraged lower than males (LMM, *F*_(1,1499)_ = 135.29, *P* < 0.001; Fig. 4C), without significant interactions between sex and social unit (LMM, *F*_(1,1499)_ = 1.44, *P* = 0.23; Fig. 4C).

### Overwinter survival

**Figure 5:**
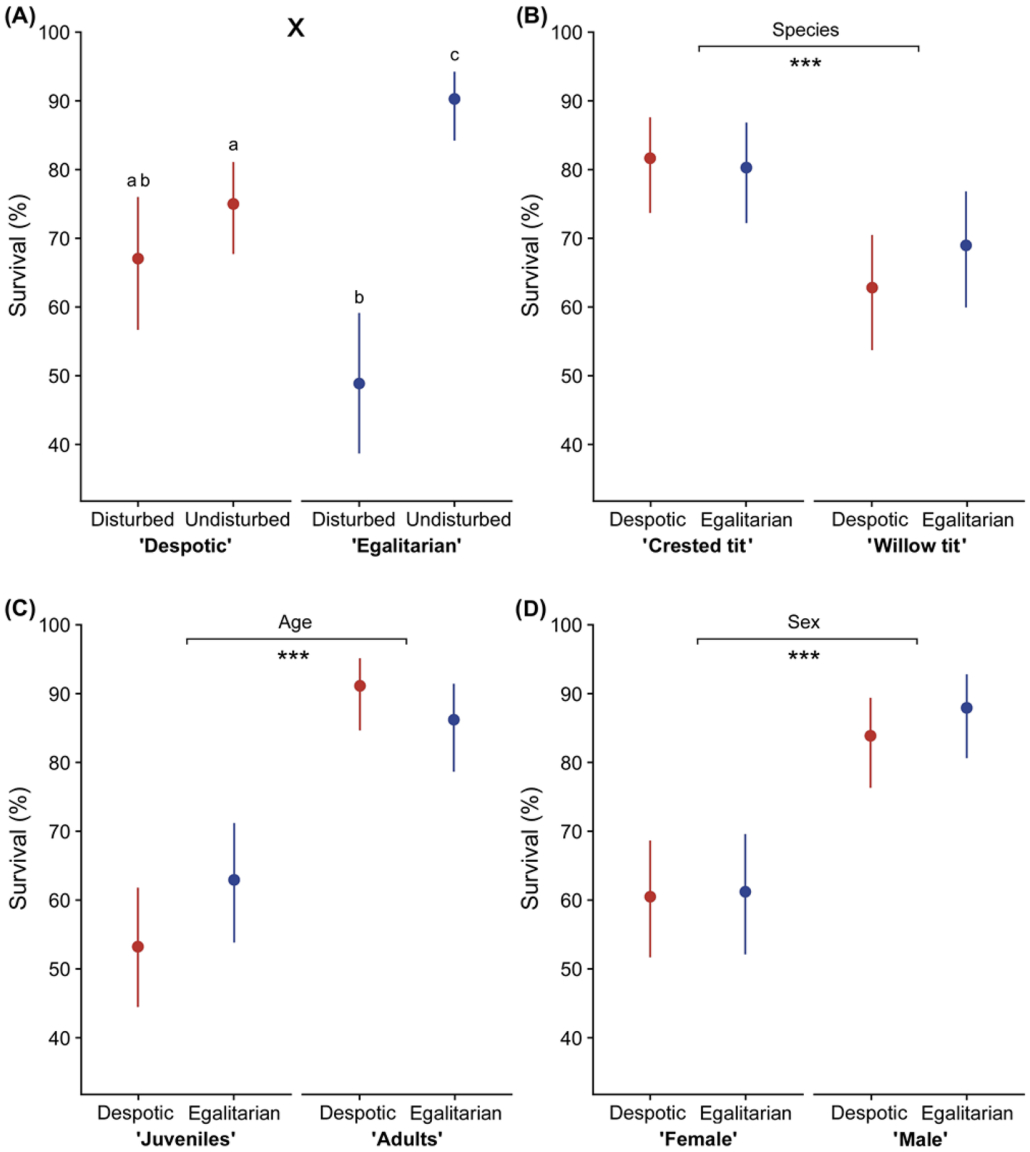
Winter survival percentage (±95%CI) of crested tits and willow tits of despotic and egalitarian mixed-species groups *vs* habitat types (**A**), species (**B**), age (**C**), sex (**D**). Letters indicate significant differences (Tukey’s tests, *p* < 0.05); X indicates significant interaction between dominance systems and habitat type. Asterisks indicate significant main effects (***P < 0.001).

We found that the survival of crested and willow tits differed significantly between the disturbed and undisturbed habitats (GLM, χ^2^ = 17.37, *P* < 0.001). Birds had higher survival in the undisturbed habitats (82.2 ± 4.2%, proportion*100 ± 95% CI, *n* = 304) than in the disturbed habitats (58.0 ± 7.2%, *n* = 176). The main effect of social unit (despotic vs. egalitarian group) on survival was non-significant (GLM, χ^2^ = 0.57, *P* = 0.45). However, there was significant interaction between habitat and type of social unit (GLM, χ^2^ = 14.07, *P* < 0.001; all other interactions (including year) were non-significant, GLM, *P*s > 0.05). Survival for both species was highest for egalitarian groups in undisturbed habitats (Fig. 5A). In the egalitarian groups, both species of tits survived better in undisturbed habitats than in the disturbed habitats (Tukey’s test, *P* < 0.05, Fig. 5A). In the despotic groups, there was no significant difference in survival between the species, habitat types and years (1993–2015 vs. 2016–2018; Tukey’s test, *P* > 0.05). Nevertheless, both species of tits in despotic groups survived better in undisturbed habitats than the tits of egalitarian groups in disturbed habitats (Tukey’s tests, *P* < 0.05; Fig. 5A). There was no significant difference in survival between despotic and egalitarian groups in disturbed habitats (Tukey’s test, *P* > 0.05, Fig. 5A). Overall, crested tits were better survivors (80.8 ± 4.9%, *n* = 240) than willow tits (65.8 ± 6.0%, *n* = 240; GLM, χ^2^ = 19.6, *P* < 0.001) (Fig. 5B). Adults survived better (88.8 ± 3.9%, *n* = 240) than juveniles (57.9 ± 6.2%, *n* = 240; GLM, χ^2^ = 54.38, *P* < 0.001; Fig. 5C) and males survived better (85.8 ± 4.4%, *n* = 240) than females (60.8 ± 6.1%, *n* = 240; GLM, χ^2^ = 41.33, *P* < 0.001; Fig. 5D).

## Discussion

### Friendlier groups are safer for subordinates

Existing data on the advantages of conspecific versus heterospecific groups provide limited evidence that mixed-species groups offer an advantage for subordinate individuals in harsh winter climates at high latitudes^22^. Here we show that social organization within mixed-species groups can significantly influence the magnitude of the survival advantage for the participating individuals. Membership in egalitarian mixed-species groups offered better chances of survival for subordinate individuals of both tit species in winter (Fig. 5). The large sample size and the broad temporal scope of our study involving consistent observations of many groups over 24 years (including 12 winters of data) clearly show that the appearance of egalitarian groups with distinct social structures in our study region is not anomalous, just rare. Another advantage of our data set is the 100% accuracy of sex and age identification on flocks with 100% of individuals uniquely marked. We found that despotic dominance structure was associated not only with lower subordinate survival but with the hypothesized intervening factors: greater subordinate fattening, increased use of risky forage sites by subordinates, higher levels of intragroup aggression and more frequent group fissions (Figs. 1–4).

Previous work on mixed-species avian groups has shown that group membership can affect participants’ fitness. For example, a species that follows parids in winter groups and is dominant over them had better body condition in mixed groups than when the nuclear parid species was experimentally removed^46^. Obligate members of tropical mixed-species avian groups also show higher survival than solitarily living species^47^. Yet solely focusing on species-level interactions in flocks may miss differences among individuals of a species in how they can benefit from mixed-species groups^48^.

Several studies have described differences in behavior in mixed groups between different sexes of a species, or individuals of varying rank^20,48,49^. Hino^50^, working with unmarked birds, found that apparently subordinate birds (judged by aggressive interactions at the time of the observation) benefitted more from mixed-species groups than dominants, a finding that we replicated here. This work and our findings emphasize the idea that one kind of benefit of mixed-species groups is the relaxation of intraspecific social forces, allowing close interaction between individuals with reduced competition. In other examples, mixed-species grouping can allow sociality among species that are highly territorial and hence do not group with conspecifics^51^, or heterospecifics can present less mating competition than conspecifics in mixed-species leks^52^. Thus, this work supports other studies in demonstrating that inter- and intraspecific social forces interact within groups, and are both important to the structure and overall benefits of mixed-species groups^19^.

Dominance hierarchies and variation in aggressiveness among participants is characteristic of animal social groups^1,53^, and can often be predicted across taxa based on the type and distribution of food resources that members of a social group use. At one extreme, herbivorous primates can exhibit egalitarian social dynamics because their food is highly dispersed, equally available to all group members and cannot be monopolized by individuals through (aggressive) contest competition. Egalitarian single-species primate groups also arise around highly clumped food concentrations under low predation risk^7,54^. In our system, however, the occurrence of both despotism and egalitarianism must be explained in the context of dispersed but rapidly depletable resources. This creates both inter- and intragroup competition, a situation thought to encourage despotic hierarchies in parid groups.

### Drivers and limits on aggression in parid groups

Extra food improves the winter survival of birds, but food resources are finite for wintering tits^23,25–27^. The food of willow tits and crested tits is dispersed and non-dividable because small arthropods and seeds constitute the largest proportion of their diet^28,55,56^. Willow tits, crested tits and other temperate parids cache their food, saving enormous amounts for later consumption^57^. Food caches, in turn, define clumped concentrations of food, and food clusters are distributed across winter group territories^57,58^. Frequent cache robbing by groupmates or members of neighboring groups affects a considerable proportion of food stored by individuals^59^. Therefore, caching favors despotism within groups, where dominant individuals can have free access to others’ caches under contest competition. Moreover, the group’s collective caches represent a shared resource. Groups of caching species vigorously defend their territories against neighbor groups stealing the resources, involving all group members in border skirmishes^37,60,61^. At the same time, daily food-gathering of dispersed resources under threat of predation counterbalances aggressive competition by enforcing more peaceful scramble competition as the mixed-species group moves around its territory gathering food to eat and cache.

The overwhelming majority of permanent members in territorial food hoarding groups of parids join the groups soon after juvenile birds fledge from their nests^62,63^. Early establishment as a permanent member of the closed groups of territorial parids has survival value as new members are recruited from the bottom of the rank order and contest competition for those positions is high^64^. Besides contests over food, parids can fight for safe foraging microsites within the groups’s foraging niche. Dominant individuals aggressively expel subordinates from the safer foraging sites which are crucial to the winter survival of parids^28,55^ (Fig. 1). Thus, although the food of crested and willow tits consists of small, non-dividable items, food hoarding dynamics, competition for group membership and intense winter predation risk encourage despotic dominance hierarchies in these groups.

Therefore, the existence of groups with egalitarian hierarchies—peaceful enough to enhance subordinate survival via reduced winter fattening, to improve access to safer foraging sites and to reduce subgroup fission—begs explanation. We consider two ways in which such hierarchies could come about: via (1) clumping of superabundant resources within group territories, thus reducing the need for contests over food and safe foraging sites, or (2) individual variation in dominance (arising via ontogenetic or heritable traits; see below). Parids are opportunistic in winter and the carcasses of moose, deer and wild boar represent a massive concentration of high-quality winter food that they can use. Carcasses can attract parid groups from 3–4 nearby territories and we have observed the local groups to defend carcasses against neighboring intruder groups. This kind of resource distribution, however, is very rare in our study area and was not observed in any of the territories of either despotic or egalitarian groups. Thus, concentrated superabundant food distribution cannot explain this pattern of sociality in parids as it does in some primates^54^.

Social learning is one more factor that has the potential to facilitate the existence of egalitarian groups. Social learning affects the exploitation of food patches and augments social attraction^65^. Although the phenomenon of social learning is considered to occur between individuals of the same species^66^, it would be important to study its role also in the multi-species context.

### High-ranking willow tit males define egalitarian groups

The defining feature of egalitarian groups was the occurrence of adult willow tit males in the second position within the multi-species dominance hierarchy rather than their usual fourth position, as in despotic groups. In turn, high-ranking willow tit males decrease aggression towards willow tit females by protecting them from lower-ranking crested tits^62^. Importantly, this feature of the egalitarian dominance hierarchy was not linked to larger size of adult willow tit males, suggesting physical strength was not the only factor that improved their dominance ranking. Adult willow tit males were, however, of lower BMI in egalitarian groups than in despotic ones. If this reflects better physical condition arising intrinsically (see below), we suggest that better body condition in adult willow tit males could be a cause (as opposed to a result) of their high ranking and the consequent difference in the organization of the groups. In contrast, it is most reasonable (given the presence of a high-ranking willow tit male in egalitarian groups) that lower BMI in associated juvenile and adult willow tit females would result from the better protection from aggression by the higher-ranking willow tit male^34^.

Two other potential causes of high-ranking arising intrinsically in these willow tits include learned and/or inherited aggressive behavior. In some species, early experience determines if an animal will be dominant or subordinate^67–69^. In birds, for example, older offspring usually have an advantage over younger siblings, but early experiences during contests can also define the overall aggressiveness of an individual. Winning one fight boosts the winner’s aggressiveness and willingness to fight in subsequent contests, potentially even with larger individuals^70^. Therefore, willow tits may rise to dominant spots due, in part, to their winning experiences during development, as these can affect social behavior throughout the individual’s life^71^.

We hypothesize that if an adult willow tit male acquired its first social experience in an egalitarian mixed-species group, this individual would claim its priority to the contested resources based on the winner-loser effect. This is likely in parid groups because adult willow and crested tits participate in mixed-species feeding associations even during the breeding season, and offspring join mixed-species groups after they fledge^72^. Moreover, familiarity of an adult willow tit male within a territory (via early establishment) could give him a “home field” advantage in dominance contests over newly immigrated crested tits^73^.

Therefore, besides the effects of heritability, some combination of ontogenetic winner-loser and environmental effects could cause a high-ranking willow tit male phenotype. Secondly, willow tit personality variation may occasionally produce especially bold aggressive males who naturally occupy a higher dominance position^74^. The reverse may also be true: aggressiveness of the top crested tit males and females may be variable and egalitarian group hierarchies may be reversed by especially non-aggressive crested tit males^75^. Natural frequencies of bold-aggressive phenotypes in both populations of tit species may determine the frequency of egalitarian groups.

### Habitat quality and survival

Finally, the general mechanism explaining the existence of relatively rare egalitarian mixed groups (consisting of ~19% of the groups in our study system) appears to be influenced also by habitat quality. The advantages of egalitarian social structure for subordinates was only the case in mature coniferous forests not affected by measures of modern forestry. In contrast, members of despotic groups showed a tendency to survive better than individuals in egalitarian groups in deteriorated areas. One limitation of interpreting this finding as driven solely by forest quality is that the data on disturbed habitats were collected during a much more limited time period (two years) than for the undisturbed habitats (24 years). Although we cannot rule out the existence of confounding variables that may arise from this discrepancy in study periods, the edge effect between the forest and the adjacent clear-cut area does make birds more exposed to predators and affects the availability of food resources, supporting our interpretation of the survival results as being driven by habitat quality. Poorer habitat quality may raise the intensity of competition for food and increase the number of aggressive interactions among group members, leading to far fewer benefits of interspecific grouping^76^.

## Conclusions

Group living animals show great variation in their social structure, from those with rigid linear dominance hierarchies to those that are more egalitarian. We show that one change in an interspecific dominance hierarchy in mixed-species tit groups, where a willow tit adult male ranks second rather than fourth, dramatically alters the fitness (survival) of these groups. We conclude that more egalitarian parid societies require more stable and predictable ecological resources and conditions, which is not possible in fragmented forest areas.

At the same time, we show that egalitarian groups remain a minority even in a mature forest (<20% of groups), so their prevalence must be limited either by some environmental (e.g., variation in the stability of resources within mature forests) or behavioral factors (e.g., limitations on how many willow tit males can hold the second place in the dominance hierarchy). In the future, experimental manipulation of food availability and distribution could more fully determine the role of habitat quality and personality-related factors in the establishment and maintenance of the two social systems and the associated winter survival patterns in mixed-species groups of willow and crested tits.

## Methods

### Study site

The main part of the study was carried out in an 80–110 year-old coniferous forest dominated by Scots pine *Pinus sylvestris* and Norway spruce *Picea abies* near the town of Krāslava (55°87′N, 27°19′E) in southeastern Latvia over a period of 24 years. Data were collected during twelve winters (1993/1994, 1995/1996, 1996/1997, 1997/1998, 2000/2001, 2004/2005, 2006/2007, 2010/2011, 2012/2013, 2013/2014, 2016/2017 and 2017/2018) between October and the beginning of March. Significant increases in clear-cut areas occurred in our study site since 2006, with a corresponding decline of old forest patch connectivity^77^. While the study between 1993 and 2017 was done in forests unaffected by forestry^78^, in winters 2016/2017 and 2017/2018 we also observed winter survival of birds in sites where forests were cut down (55°89′N, 27°26′E). Areas that were clear cut appeared just on the border of eleven egalitarian groups (88 birds) and eleven despotic groups (88 birds). The body mass data were collected annually at the end of December through to January during which daily ambient temperatures varied between 0 °C and −20 °C. This temperature range is typical for most winters in the study area. The data can therefore be compared across the study periods.

### Birds

In total, we studied 60 mixed species groups (31 ‘despotic’ and 29 ‘egalitarian’ groups) of willow tits (*n* = 240) and crested tits (*n* = 240) (Supplementary information). Winter groups typically consist of permanent members that are often joined by “floaters”, juvenile individuals that travel between the groups within a restricted area^79,80^. In this study, we considered only permanent group members: all the mixed-species groups contained equal numbers of crested tits (mean number of individuals = 4.0, *SD* = 0) and willow tits (mean number of individuals = 4.0, *SD* = 0). They had been sexed and aged (as adult or juvenile; juveniles are individuals that were born in the year of the study, while adults are individuals that have already had a reproductive season in the calendar year) either in the previous breeding seasons or in September of each study year. The birds were trapped by mist nets (Ecotone, Sopot, Poland) at temporary feeders baited with sunflower seeds. Each bird was banded with metal and individually recognizable plastic rings. The shape of the rectrices of willow and crested tits^81^ and the color of the iris of crested tits^82,83^ were used to determine age. A variety of methods allowed us to determine the sex of individuals in the groups, including: (a) sexual dimorphism in wing and tarsus length^26^; (b) wing length variations based on the methods of Vinogradova *et al*.^84^; willow tits with wings <61 mm = females, and willow tits with wings >67 mm were considered males; and (c) observations of individual willow and crested tits outside the wintering season. Observed fights and territorial behavior, singing behavior and parental roles during the breeding season were used to determine sex of those individuals with overlapping biometrical parameters. DNA sexing was done only in 2016/2017 and 2017/2018. The samples were collected by buccal swabs and DNA was obtained by using Chelex DNA-extraction according to Adam *et al*.^85^. This non-invasive procedure is based on the RRR (replacement, reduction, refinement) principle^86^ which facilitates more ethical use of animals in experimental testing. Behavioral and morphological sexing was 100% correct in birds that were DNA-sexed. Therefore, we assume that our previous assignments of sex were correct, despite the lack of DNA testing prior to 2016/2017. Only groups with all individuals properly sexed were included in the analyses.

Importantly, as soon as an egalitarian group was identified, we started conducting comparative research on one or two despotic groups located in the same forest. Territories of these groups had a common border. This allowed us to minimize confounding factors related to the quality of territories, forest type, age and microclimate. We studied between two to three egalitarian groups each season. In this study, we have never sampled the same groups in the next wintering season.

### Dominance hierarchies

Dominance order was measured within each group by observing pairwise interactions between birds at temporary feeders filled with sunflower seeds and fat. The food was provided during observation hours only and the birds were trained to come to the feeders in the territories when hearing a specific auditory signal. We recorded at least 150 aggressive within-group encounters per group (*n* = 60; total 9256 aggressive encounters). To determine individual rank, we followed the procedures of Koivula and Orell^26^. The dominant won more interactions than the subordinate within each dyad (two-tailed sign-test, *P* < 0.001). The number of aggressive interactions within and between both species was counted annually in January when general level of aggression was considered to be lowest (personal obs.). Each group with free-ranging birds was followed for 30 min between 11:00 and 14:30 when the birds foraged in the canopy. Each group was observed at least 6 times, no more than once per day.

In 31 ‘despotic’ groups, there was a clear dominance hierarchy: crested tits were the dominants and willow tits the subordinates, with adult willow tit males dominating over juvenile crested tit females as the only exception. In contrast, in 29 ‘egalitarian’ mixed-species groups we found that adult willow tit males dominated juvenile crested tit females, juvenile crested tit males and adult crested tit females, so that willow tit adult males were the second-ranking individuals behind adult crested tit males. The proportion of 31 despotic and 29 egalitarian groups does not reflect the natural occurrence of these two social systems in willow and crested tits because we studied more groups than the reported 60 to find those 29 egalitarian ones. In total, we investigated 156 mixed-species groups, demonstrating that ‘egalitarianism’ (18.59% of all inspected groups) is a rather rare phenomenon in willow and crested tits.

### Body mass and body reserves

When examining the feeding efficiency of dominant and subordinate individuals of each species, we used repeated weighings on different days for all members of a group. The body mass of birds was measured when they landed on an electronic balance (Sartorius AZ212, Göttingen, Germany; precision of 0.1 g) located at the feeder. The data were collected at the last hour of the birds’ daily activity period in the beginning of each winter. Only one measurement was taken per bird in a day. In total, we obtained 10 weighings as the mean number of measurements per individual crested tit and 11 per individual willow tit per fortnight. Differences between the dominant and subordinate species in the way evening body mass varied among individuals were tested by using relative body mass. The evening body mass was transformed into the body mass index (BMI) by taking the ratio of body mass to wing length, scaling body reserves according to bird size^87^. BMI was calculated by dividing body mass by the third power of wing length (body mass/(wing length × 10^3^)). Extra fat increases wing load, which negatively affects maneuverability and the speed of escape behavior^36^. Hence, wing length is of biological significance for subcutaneous fat reserves and, ultimately, predation risk^35,88,89^.

### Group cohesion

To record the coherence of the groups, each group was followed for one hour during the first hour of the birds’ daily activity and for one hour at noon. We recorded the proportion of time that willow tits spent together with crested tits in the same social unit. As soon as individuals of either species split into two units and moved away farther than 100 m from each other for more than 10 min, the mixed-species group was considered as being split. We expected that willow tits would tend to leave crested tits more often at noon, a period when the level of intragroup aggression is high while the level of predation risk is reduced and when food availability is most likely increased due to higher daytime temperatures^90,91^. We measured the closest distance between two monospecific subgroups by a Nikon Forestry Pro Laser Rangefinder (Nikon Vision Company, Tokyo, Japan).

### Foraging sites in the canopy

The use of foraging sites was observed between 10:30 and 14:30. For each observation we estimated the foraging height of the focal bird. Foraging heights were expressed as proportions of canopy height divided into four categories^24,91^. To avoid discovery bias, foraging site use was recorded with a delay of 15 s between the identification of a bird and the recording of its position in the tree. Such a time delay appeared to be sufficient to avoid discovery bias by ensuring that the activity recorded had a chance to differ from the one noted when the bird was first sighted^24,28^. All members of a group were observed on the same day. We recorded up to five observations of the same individual with an interval of at least 5 min between the observations. We averaged the foraging heights for every individual for each day and observed each individual for 8–10 days.

### Winter survival

We estimated the survival of crested and willow tits at the end of February through to the beginning of March. We checked for the presence/absence of group members for 4–5 days by observing color-banded individuals with binoculars (a 10x magnification was used) at bird feeders baited with sunflower seeds^92–94^. The birds that disappeared in the course of winter but were found again outside the study area as members of other mixed-species groups in winter/early spring (*n* = 9), or were found in spring/summer near their nests (*n* = 8) or together with their offspring (*n* = 1), were included in the analyses as survivors.

### Predators and predation risk

The European sparrowhawk (*Accipiter nisus*) and the pygmy owl (*Glaucidium passerinum*) are the principal predators of the wintering tits in this region^24,91^, representing the main cause of parid winter mortality. We observed these airborne predators daily in the study area. Sparrowhawks attack their prey while chasing them in sparse lower parts of the coniferous canopy where the predator can reach its maximum speed. Pygmy owls sometimes hunt actively by moving from perch to perch with short flights. However, they mainly hunt as “sit-and-wait” predators that perform their attacks from above by swooping down on the prey^88^. The risk of being attacked and killed by a pygmy owl or a sparrowhawk is a function of the relative canopy height and distance from the trunk^20,24,90,95^ (Fig. 1), with peripheral and lower parts of the canopy providing less protection against these predators.

### Statistical analyses

Analyses were carried out in R, v. 3.3.2 and Statistica 8.0 for Windows (StatSoft Inc., Tulsa, OK, USA). We used a binomial generalized mixed effects model with group ID as a random effect to compare winter survival of birds. Social unit (‘despotic’, ‘egalitarian’), habitat (disturbed, undisturbed), species (crested tit, willow tit), sex and age (juvenile, adult) were used as fixed factors to assess the differences in winter survival. Confidence intervals for binomial probabilities were calculated according to Agresti and Coull^96^. We used ANOVA and a linear mixed model (LMM) with species, social unit, sex and age as fixed factors to assess the differences in relative feeding height. Individual bird identity and group identity nested within group were used as random effects. We also included all two-way interactions between the factors. If a two-way interaction was significant, a post hoc test (Tukey) was applied. LMMs were fit using the nlme package^97^ as implemented in R 3.3.2^98^. We used Spearman’s correlation to measure the relationship between the BMI of individuals of the two tit species and their dominance rank. We also used *t*-tests and Mann-Whitney *U* tests to determine whether two groups were different from each other.

### Ethical note

We confirm that all methods were carried out in accordance with relevant guidelines and regulations of the Republic of Latvia. All research protocols were approved by the Latvian Nature Conservation Agency and Food and Veterinary Service of the Republic of Latvia.

## Supplementary information

Supplementary information

## Data Availability

All data sets are available upon request.

## References

[1] Krause, J. & Ruxton, G. D. *Living in groups* (Oxford University Press, 2002).

[2] Goodale E (2015). The structure of mixed-species bird flocks, and their response to anthropogenic disturbance, with special reference to East Asia. Avian. Res..

[3] Goodale, E., Beuchamp, G. & Ruxton, G. D. *Mixed-Species Groups of Animals: Behavior, Community Structure, and Conservation*. (Academic Press, 2017).

[4] Eguchi K, Yamagishi S, Randrianasolo V (1993). The composition and foraging behaviour of mixed-species flocks of forest-living birds in Madagascar. Ibis.

[5] Goodale E (2009). Regional variation in the composition and structure of mixed-species bird flocks in the Western Ghats and Sri Lanka. Curr. Sci. India.

[6] Sridhar H (2012). Positive relationships between association strength and phenotypic similarity characterize the assembly of mixed-species bird flocks worldwide. Am. Nat..

[7] Hermann, H. R. Dominance and Aggression in Humans and Other Animals: The Great Game of Life (Academic Press, 2017).

[8] Pulliam HR (1973). On the advantages of flocking. J. Theor. Biol..

[9] Elgar MA (1989). Predator vigilance and group size in mammals and birds: a critical review of the empirical evidence. Biol. Rev..

[10] Lima S (1995). Back to the basics of anti-predatory vigilance: the group-size effect. Anim. Behav..

[11] Olson Randal S., Haley Patrick B., Dyer Fred C., Adami Christoph (2015). Exploring the evolution of a trade-off between vigilance and foraging in group-living organisms. Royal Society Open Science.

[12] Ekman J (1989). Ecology of non-breeding social systems of Parus. Wilson Bull..

[13] Matthysen E (1990). Nonbreeding social organization in Parus. Curr Ornithol..

[14] Beauchamp, G. *Social predation: How group living benefits predators and prey* (Academic Press, 2014).

[15] MacArthur R, Levins R (1967). The limiting similarity, convergence, and divergence of coexisting species. Am. Nat..

[16] Dhondt, A. A. *Interspecific Competition in Birds* (Oxford University Press, 2011).

[17] Gil MA, Emberts Z, Jones H, St Mary CM (2017). Social information on fear and food drives animal grouping and fitness. Am. Nat..

[18] Sridhar H, Guttal V (2018). Friendship across species borders: factors that facilitate and constrain heterospecific sociality. Phil. Trans. Roy. Soc..

[19] Krams I, Krama T, Freeberg TM, Kullberg C, Lucas JR (2012). Linking social complexity and vocal complexity: a parid perspective. Phil. Trans. Roy. Soc. B.

[20] Suhonen J (1993). Risk of predation and foraging sites of individuals in mixed-species tit flocks. Anim. Behav..

[21] Suhonen J (1993). Predation risk influences the use of foraging sites by tits. Ecology.

[22] Hogstad O (1988). The influence of energy stress on social organization and behaviour of willow tits Parus montanus. Fauna Norv. Ser. C..

[23] Hogstad O (1988). Advantages of social foraging of willow tits Parus montanus. Ibis.

[24] Krams IA (1996). Predation risk and shifts of foraging sites in mixed willow and crested tit flocks. J. Avian Biol..

[25] Ekman J, Cederholm G, Askenmo C (1981). Spacing and survival in winter groups of willow tit Parus montanus Conrad and crested tit Parus cristatus L. – a removal study. J Anim Ecol.

[26] Koivula K, Orell M (1988). Social rank and winter survival in the willow tit Parus montanus. Ornis Fenn.

[27] Koivula K, Orell M, Rytkönen S (1996). Winter survival and breeding success of dominant and subordinate willow tits Parus montanus. Ibis.

[28] Ekman J (1986). Tree use and predator vulnerability of wintering passerines. Ornis Scand..

[29] Hogstad O (1989). Subordination in mixed-age bird flocks-a removal study. Ibis.

[30] Lahti K (1998). Social dominance and survival in flocking passerine birds: a review with an emphasis on the willow tit Parus montanus. Ornis Fenn..

[31] Rodríguez A, Jansson G, Andrén H (2007). Composition of an avian guild in spatially structured habitats supports a competition–colonization trade-off. Proc. Roy. Soc. B..

[32] Speakman JR (2018). The evolution of body fatness: trading off disease and predation risk. J. Exp. Biol..

[33] Ekman JB, Hake MK (1990). Monitoring starvation risk: adjustments of body reserves in greenfinches (Carduelis chloris L.) during periods of unpredictable foraging success. Behav. Ecol..

[34] Krams I (1998). Rank-dependent fattening strategies of willow tit Parus montanus and crested tit P. cristatus mixed flock members. Ornis Fenn..

[35] Witter MS, Cuthill IC, Bonser RHC (1994). Experimental investigations of mass-dependent predation risk in the European starling, Sturnus vulgaris. Anim Behav.

[36] Krams I (2002). Mass-dependent take-off ability in wintering great tits (Parus major): comparison of top-ranked adult males and subordinate juvenile females. Behav. Ecol. Sociobiol..

[37] Hogstad O (1987). Social rank in winter flocks of willow tits Parus montanus. Ibis.

[38] Freeberg TM (2006). Social complexity can drive vocal complexity: group size influences vocal information in Carolina chickadees. Psychol. Sci..

[39] Grabowska-Zhang AM, Sheldon BC, Hinde CA (2012). Long-term familiarity promotes joining in neighbour nest defence. Biol. Lett..

[40] Goodale E, Beauchamp G, Magrath R, Nieh JC, Ruxton GD (2010). Interspecific information transfer influences animal community structure. Trends Ecol. Evol..

[41] Firth J, Sheldon BC (2016). Social carry-over effects underpin trans-seasonally linked structure in a wild bird population. Ecol. Lett..

[42] Freeberg TM, Dunbar RIM, Ord TJ (2012). Social complexity as a proximate and ultimate factor in communicative complexity. Phil. Trans. Roy. Soc. B..

[43] Chazdon RL (2009). The potential for species conservation in tropical secondary forests. Conserv. Biol..

[44] Griesser M, Nystrand M (2009). Vigilance and predation of a forest-living bird species depend on large-scale habitat structure. Behav. Ecol..

[45] Barlow J (2007). Quantifying the biodiversity value of tropical primary, secondary, and plantation forests. Proc. Natl. Acad. Sci. USA.

[46] Dolby AS, Grubb TC (1998). Benefits to satellite members in mixed-species foraging groups: an experimental analysis. Anim. Behav..

[47] Jullien M, Clobert J (2000). The survival value of flocking in neotropical birds: reality or fiction?. Ecology.

[48] Farine DR, Garroway CJ, Sheldon BC (2012). Social network analysis of mixed-species flocks: Exploring the structure and evolution of interspecific social behaviour. Anim. Behav..

[49] Farine DR, Milburn PJ (2013). Social organisation of thornbill-dominated mixed-species flocks using social network analysis. Behav. Ecol. Sociobiol..

[50] Hino T (2000). Intraspecific differences in benefits from feeding in mixed-species flocks. J. Avian Biol..

[51] Marra PP, Sherry TW, Holmes RT (1993). Territorial exclusion by a long-distance migrant warbler in Jamaica: a removal experiment with American redstarts (Setophaga ruticilla). Auk.

[52] Gibson RM, Aspbury AS, McDaniel LL (2002). Active formation of mixed-species grouse leks: a role for predation in lek evolution?. Proc. Roy. Soc. B..

[53] Weise CM, Meyer JR (1979). Juvenile dispersal and development of site-fidelity in the black-capped chickadee. Auk.

[54] Strier, K. B. *Primate Behavioral Ecology* (Routledge, 2016).

[55] Jansson C, von Brömssen A (1981). Winter decline of spiders and insects in spruce Picea abies and its relation to predation by birds. Holarct Ecol..

[56] Suhonen J, Alatalo RV, Carlson A, Höglund J (1992). Food resource distribution and the organization of the Parus guild in a spruce forest. Ornis Scand.

[57] Pravosudov VV (1986). Individual differences in foraging and storing behaviour in Siberian tit Parus cinctus Bodd. and willow tit Parus montanus Bald. Soviet. J. Ecol..

[58] Sherry D, Avery DM, Stevens A (1982). The spacing of stored food by marsh tits. Z Tierpsychol.

[59] Stevens TA, Krebs JR (1986). Retrieval of stored see& by marsh its (Parus palustris) in the field. Ibis.

[60] Inki K, Suhonen J (1993). Characteristics of cache sites most likely to be robbed by willow tits (Parus montanus). Condor.

[61] Smith, S. M. The Black-capped Chickadee: Behavioral Ecology and Natural History (Cornell University Press, 1991).

[62] Krams I (2000). Long-range call use in dominance-structured crested tit Parus cristatus winter groups. J. Avian Biol..

[63] Ward, A. & Webster, M. Sociality: The Behaviour of Group-Living Animals (Springer, 2016).

[64] Nilsson JA, Smith H (1988). Effects of dispersal date on winter flock establishment and social dominance in marsh tits Parus palustris. J. Anim. Ecol..

[65] Tóth Z, Tuliozi B, Baldan D, Hoi H, Griggio M (2017). The effect of social connections on the discovery of multiple hidden food patches in a bird species. Sci. Rep..

[66] Loukola OJ, Seppänen J-T, Krams I, Torvinen SS, Forsman JT (2014). Observed fitness may affect niche overlap in competing species via selective social information use. Am. Nat..

[67] Mineka S, Gunnar M, Champoux M (1986). Control and early socioemotional development: Infant rhesus monkeys reared in controllable versus uncontrollable environments. Child Developm..

[68] Vellucci, S.V. Primate social behavior: anxiety or depression in Psychopharmacology of Allxiolylics and Anlidepressants (ed. File, S. E.) 83–105, (Pergamon Press, 1991).

[69] Carlson BA (2017). Early life experiences have complex and long-lasting effects on behaviour. Proc. Natl. Acad. Sci. USA.

[70] Dugatkin LA (1997). Winner and loser effects and the structure of dominance hierarchies. Behav. Ecol..

[71] Drummond H, Osorno JL (1992). Training siblings to be submissive losers: dominance between booby nestlings. Anim. Behav..

[72] Mönkkönen M, Forsman JT, Helle P (1996). Mixed species foraging aggregations and heterospecific attraction in boreal bird communities. Oikos.

[73] Fuxjager MJ (2010). Winning territorial disputes selectively enhances androgen sensitivity in neural pathways related to motivation and social aggression. Proc. Natl. Acad. Sci. USA.

[74] Colléter M, Brown C (2011). Personality traits predict hierarchy rank in male rainbowfish. Anim Behav.

[75] Kurvers RHJM (2009). Personality differences explain leadership in barnacle geese. Anim. Behav..

[76] Gavrilets S (2012). On the evolutionary origins of the egalitarian syndrome. Proc. Natl. Acad. Sci. USA.

[77] Rendenieks Z, Nikodemus O, Brūmelis G (2015). Dynamics in forest patterns during times of forest policy changes in Latvia. European. J. Forest. Res..

[78] Rytkönen S, Krams I (2003). Does foraging behaviour explain the poor breeding success of great tits Parus major in northern Europe?. J. Avian Biol..

[79] Smith SM (1984). Flock switching in chickadees: why be a winter floater?. Am. Nat..

[80] Hogstad Olav (2015). Social behaviour in the non-breeding season in Great Tits Parus major and Willow Tits Poecile montanus: differences in juvenile birds’ route to territorial ownership, and pair-bond stability and mate protection in adults. Ornis Norvegica.

[81] Laaksonen M, Lehikoinen E (1976). Age determinations of willow and crested tit Parus montanus and P. cristatus. Ornis Fenn..

[82] Svensson, L. *Identification Guide to European Passerines* (BTO, 2016).

[83] Lens L, Dhondt AA (1992). Variation in coherence of crested tit winter flocks: an example of multivariative optimization. Acta Oecol..

[84] Vinogradova, N. V., Dolnik, V. R., Efremov, V. D., Paevskij, V. A. Identification of Sex and Age of Passerine Birds of the USSR Fauna (Nauka, 1976).

[85] Adam I, Scharff C, Honarmand M (2014). Who is who? Non-invasive methods to individually sex and mark altricial chicks. J. Visual Exp..

[86] Russell, W. M. S. & Burch, R. L. *The Principles of Humane Experimental Technique. Special edition published by Universities Federation for Animal Welfare (UFAW), 1992* (Methuen & Co, 1959).

[87] Summers RW (1988). The use of linear measurements when comparing masses. Bird Study.

[88] McNamara JM, Houston AI (1990). The value of fat reserves and the trade-off between starvation and predation. Acta Biotheor..

[89] Pravosudov VV (1999). Social dominance and energy reserves in wintering woodland birds. Condor.

[90] Kullberg C (1995). Strategy of pygmy owl while hunting avian and mammalian prey. Ornis Fenn..

[91] Krams I (2000). Length of feeding day and body weight of great tits in a single-and a two-predator environment. Behav. Ecol. Sociobiol..

[92] Krams IA, Krams T, Černihovičs J (2001). Selection of foraging sites in mixed willow and crested tit flocks: rank-dependent strategies. Ornis Fenn..

[93] Krama T (2015). Intensity of haemosporidian infection of parids positively correlates with proximity to water-bodies, but negatively with host survival. J. Ornithol..

[94] Cīrule D (2017). Habitat quality affects stress responses and survival in a bird wintering under extremely low ambient temperatures. Science of Nature.

[95] Kullberg C, Ekman J (2000). Does predation maintain tit community diversity?. Oikos.

[96] Agresti A, Coull BA (1998). Approximate is better than “exact” for interval estimation of binomial proportions. Am. Statistic.

[97] Pinheiro, J., Bates, D., DebRoy, S., Sarkar, D. & R Core Team. nlme: linear and nonlinear mixed effects models. *R package version* 3.1–137, https://CRAN.R-project.org/package=nlme (2018).

[98] R Core Team. R: A language and environment for statistical computing. R Foundation for Statistical Computing, Vienna, Austria, https://www.R-project.org/ (2016).

